# Aberrant Promoter CpG Methylation Is a Mechanism for Impaired PHD3 Expression in a Diverse Set of Malignant Cells

**DOI:** 10.1371/journal.pone.0014617

**Published:** 2011-01-28

**Authors:** Trenton L. Place, Matthew P. Fitzgerald, Sujatha Venkataraman, Sabine U. Vorrink, Adam J. Case, Melissa L. T. Teoh, Frederick E. Domann

**Affiliations:** 1 Molecular and Cellular Biology Program, The University of Iowa, Iowa City, Iowa, United States of America; 2 Free Radical and Radiation Biology Program and Department of Radiation Oncology, The University of Iowa, Iowa City, Iowa, United States of America; 3 University of Colorado Denver, Pediatrics, Aurora, Colorado, United States of America; 4 Human Toxicology Program, The University of Iowa, Iowa City, Iowa, United States of America; 5 Carver College of Medicine and The Holden Comprehensive Cancer Center, The University of Iowa, Iowa City, Iowa, United States of America; Louisiana State University, United States of America

## Abstract

**Background:**

The prolyl-hydroxylase domain family of enzymes (PHD1-3) plays an important role in the cellular response to hypoxia by negatively regulating HIF-α proteins. Disruption of this process can lead to up-regulation of factors that promote tumorigenesis. We observed decreased basal expression of PHD3 in prostate cancer tissue and tumor cell lines representing diverse tissues of origin. Furthermore, some cancer lines displayed a failure of PHD3 mRNA induction when introduced to a hypoxic environment. This study explores the mechanism by which malignancies neither basally express PHD3 nor induce PHD3 under hypoxic conditions.

**Methodology/Principal Findings:**

Using bisulfite sequencing and methylated DNA enrichment procedures, we identified human *PHD3* promoter hypermethylation in prostate, breast, melanoma and renal carcinoma cell lines. In contrast, non-transformed human prostate and breast epithelial cell lines contained *PHD3* CpG islands that were unmethylated and responded normally to hypoxia by upregulating PHD3 mRNA. Only treatment of cells lines containing *PHD3* promoter hypermethylation with the demethylating drug 5-aza-2′-deoxycytidine significantly increased the expression of PHD3.

**Conclusions/Significance:**

We conclude that expression of PHD3 is silenced by aberrant CpG methylation of the *PHD3* promoter in a subset of human carcinoma cell lines of diverse origin and that this aberrant cytosine methylation status is the mechanism by which these cancer cell lines fail to upregulate PHD3 mRNA. We further show that a loss of PHD3 expression does not correlate with an increase in HIF-1α protein levels or an increase in the transcriptional activity of HIF, suggesting that loss of PHD3 may convey a selective advantage in some cancers by affecting pathway(s) other than HIF.

## Introduction

The cellular response to reduced oxygen availability (hypoxia) is controlled by a class of proteins called hypoxia-inducible factors (HIF-α). There are 3 known isoforms of HIF-1α: HIF-1α, HIF-2α and HIF-3α. HIF-1α and HIF-2α are transcription factors. HIF-3α appears to lack transcriptional activity and may play a role in negative regulation of the HIF pathway [1]. Thus, from here on, when referring to HIF-α, we are referring to only HIF1 and HIF2. Transcriptionally active HIF1 and 2 are heterodimers composed of the HIF-α subunit and aryl hydrocarbon nuclear translocator receptor (ARNT/HIF-β)HIF-1α activates the transcription of EPO, VEGF, heme oxygenase-1 and several other critical intracellular responses to hypoxia including enzymes of the glycolytic pathway [2], [3]. While less is known about HIF-2α transcriptional targets, HIF-2α appears to play a lesser role in the glycolytic response with more emphasis on EPO and VEGF transcription [4].

HIF-α mRNA levels are generally stable in cells. It is not until after translation that HIF-α is tightly controlled. During periods of normal physiological oxygen concentration, HIF-α subunits are kept at low levels by constant proteolytic degradation. First, a hydroxylation reaction is catalyzed by a family of prolyl hydroxylase domain-containing proteins (PHD/EGLN/HPH) which utilize iron, oxygen and 2-oxoglutarate as co-factors to enzymatically catalyze hydroxylation on the oxygen-dependent degradation domain (ODD) of the HIFα -subunit [5]. Hydroxylated proline residues on HIF-α are recognized by Von Hippel-Lindau (VHL) protein, an E3 ubiquitin ligase that ubiquitinates the HIF-α subunit, targeting it to the proteosome [6]. Under hypoxic conditions, HIF prolyl hydroxylase activity is decreased and HIF-1α protein accumulates. HIF-α subunits translocate to the nucleus and dimerize with the constitutively expressed ARNT subunit [7], [8]. This heterodimer acts to turn on transcription of genes involved in oxygen homeostasis and glucose metabolism [2].

Three main isoforms of HIF prolyl-hydroxylase domain containing proteins, PHD1-3, have been identified [9]. These isoforms have been reported to have different specificities for HIF-1α and HIF-2α [10], and also differ in their subcellular localization. It has been shown that PHD1 is exclusively present in cytoplasm, PHD2 is mainly located in the nucleus and PHD3 is evenly distributed in both cytoplasm and nucleus [11]. PHD2 and PHD3, however, are considered to be the major isoforms that contribute to HIF-1 and -2α degradation in cells [12], [13]. In normoxia, PHD2 is the primary enzyme that hydroxylates HIF-1α [14], whereas PHD3 has been reported to play an important role in HIF-2α hydroxylation and also in retaining cellular hydroxylation capacity in a hypoxic environment [10], [15].

In normal cells, PHD3 mRNA and protein are expressed at low levels during normoxia, but are significantly induced upon exposure to hypoxia. In contrast, PHD3 expression in a significant number of cancer cell types has been shown to be low or absent not only during normoxia, but also under hypoxic conditions [10], [16]. To date, no mechanism has been discovered to explain this defect in hypoxic inducibility. Interestingly, Hatzimichael et al. have recently demonstrated that the promoter of PHD3 is methylated in certain primary B-cell dyscrasias [17]. We had observed a decrease in PHD3 mRNA expression in human breast and prostate carcinoma cell lines, with an absence of PHD3 upregulation in response to hypoxia. Therefore, we were interested to determine whether PHD3 promoter methylation was responsible for this aberrant expression pattern. In this study, we show that the promoter region of PHD3 is methylated in representative human prostate carcinoma, melanoma, renal carcinoma and breast cancer cell lines. Furthermore, we show that neither HIF-1α protein levels nor hypoxic response through an HRE-luciferase reporter vector are compromised in PHD3 methylated compared to non-methylated cell lines. These results indicate that PHD3 promoter methylation is utilized by malignancies derived from diverse human cell types. Furthermore, these data suggest that loss of PHD3 expression may not affect the transcriptional response through the HIF pathway, leaving open the possibility that PHD3 silencing in tumors is selected through the loss of specific interactions with other cellular pathways.

## Methods

### Cell culture

Normal human prostate epithelial cells (NPrEC) were purchased from Clonetics, Lonza Inc. (Walkersville, MD) and were grown on the recommended PrEGM media supplied by Clonetics, Lonza Inc. The hTERT-HME1 cells were cultured in mammary epithelial basal medium MEGM (Lonza Inc.) at 37°C and 5% CO_2_ according to the manufacturer's instructions (Lonza Inc.). The DU 145, 22RV.1, PC-3, MDA-MB-435 (MB-435), and MCF7 cell lines were obtained from ATCC (Manassas, VA). MCF7, DU145 and MDA-MB-435 cells were cultured in Eagle's Minimum Essential Medium (MEM) supplemented with 10% fetal bovine serum (FBS). PC-3 cells were cultured in F12 medium supplemented with 10% FBS, 2 mM L-glutamine, 1 mM Na Pyruvate, and supplemented with 100 U/ml pen/strep. All cell lines were routinely maintained at 37°C in a humidified atmosphere with 5% CO_2_. Fresh media was replaced every three days while routine subculture was performed by washing with 1X PBS and detaching cells with TrypLE Express.

### Semi-quantitative RT-PCR

Total RNA was extracted from individual cell lines using RNeasy Mini Kit (Qiagen, Valencia, CA) and quantified using a NanoDrop 1000. To assess *PHD3* and GAPDH expression, 500 ng of total RNA was used for reverse transcription using a OneStep RT-PCR Kit (Qiagen). The *PHD3* forward primer is 5′-GGGCAAATACTACGTCAAGGAG-3′ and the reverse primer is 5′-AGTCTTCAGTGAGGGCAGATTC-3′. GAPDH expression was assessed using GAPDH-specific primers. PCR conditions for *PHD3* and GAPDH were the same except that 28 cycles of PCR were performed for *PHD3* analysis and 23 cycles were performed for GAPDH. The parameters used were: 95°C for 5 minutes followed by the stated number of cycles of 94°C for 1 minute; 56°C for 1 minute, and 72°C for 1 minute, ending with a final extension at 72°C for 7 minutes. The amplified products were electrophoresed on a 1% agarose gel and stained with ethidium bromide to visualize the bands.

### Quantitative real time RT-PCR

Total RNA was isolated from cells using Trizol, followed by DNAse treatment and NaOAc precipitation. The reverse transcription reaction was carried out with High-Capacity cDNA Archive Kit (Applied Biosystems, Foster City, CA). PHD3 TaqMan primer-probe was utilized from Applied Biosystems (Hs00222966_m1). The quantitative real-time PCR was set up as follows: 10 ng of RNA was used as template for each real-time PCR reaction (10 µg reaction volume); primer pairs at 0.3 µM for GAPDH with Syber Green Master Mix (Applied Biosystems). For PHD3, TaqMan universal master mix was used. The DNA polymerase was activated by heat at 95°C for 10 min followed by 40 cycles, denaturing at 95°C for 15 s, annealing and elongating at 60°C for 1 min. Data were collected with ABI PRISM 7000 sequence detection system. Data were analyzed using the ΔΔCt method.

### Western Blot analysis

Cells were immediately washed with ice cold phosphate-buffered saline (pH 7.4). Cells were lysed on the plate in 200 µl RIPA cell-lysis buffer (50 mM Tris pH 8.0, 150 mM NaCl, 0.1% SDS, 0.5% Na Deoxycholate, 1% TX-100) plus 1 mM NaF, 10 mM NaVO_4_, 10 mM PMSF, and 1/100 protease inhibitor cocktail (Sigma), immediately boiled for 2 minutes and then sonicated. SDS-polyacrylamide gels (7%, PHD3; 15% HIF1, HIF2) were used for protein electrophoresis. Proteins were electrotransferred onto nitrocellulose membranes and treated with anti HIF-1α (Abcam, Cambridge, MA) 1∶500 overnight at 4°C. Anti PHD3, NB100-139 and anti HIF-2α antibodies (Novus Biologicals, Littleton, CO) were used at 1∶500 and 1∶200 respectively overnight at 4°C. Equal protein loading was confirmed on all immunoblots using human actin antibody (Sigma, St. Louis, MO) at a dilution 1∶2000. Goat anti-rabbit IgG (BD Transduction Laboratories, San Diego, CA) was used as a secondary antibody against all primary antibodies. Bands were visualized by chemiluminescence with ECL plus reagent (Pierce, Rockford, IL) on a Typhoon FLA 7000.

### Sodium bisulfite sequencing

Genomic DNA was extracted with the use of the DNeasy Tissue Kit (Qiagen, Valencia, CA), and sodium bisulfite conversion was performed with the use of the EZ DNA Methylation Kit (Zymo Research Corporation, Orange, CA). A pair of primers was designed to amplify the PHD3 promoter of both bisulfite modified methylated and unmethylated DNA but not unmodified DNA. Nested PCR amplification on converted DNA used the following primers: outside forward: 5′-GTGTGGGATTTAGGTTTTTAAG-3′ (SB1); outside reverse:5′-CCAAATCCAACCTCATAATATATC-3′ (SB2); and nested inner primers (SB3) and (SB4) whose sequences and locations are described in detail below.

The resulting PCR products were gel-extracted with the use of the Qiagen Gel Extraction Kit, or gel digestion with β-agarase followed by EtOH precipitation, and cloned with the TOPO TA Cloning Kit (Invitrogen). Plasmid DNA was extracted with the use of the QiaPrep Spin Plasmid Miniprep Kit (Qiagen). Sequencing was performed by the sequencing core facility maintained by the University of Iowa and results were tabulated for methylation status of each of the 58 CpGs contained in the amplicons from each cell line.

### 5-Aza-dC treatment

Cells were counted, and seeded (day 0) at approximately 750,000 cells/100 mm dish. Fresh 5-Aza-dC (5 mM) was added to the dish on days 1, 3, and 5 while a control flask was left untreated. On day 5, 5-Aza-dC treated cells were split into two 60 mm dishes in media supplemented with 5 mM 5-Aza-dC. On day 6, one of each of the 60 mm dishes was placed in a hypoxia chamber and placed under 1%O_2_, 94%N_2_ and 5% CO_2_ at 37°C. On day 7, all cells were harvested with 500 µl Trizol for RNA extraction.

### Chromatin Accessibility

Chromatin accessibility experiments were conducted as previously described by Rose *et al.* [18]. The primers CA1 and CA2 were located within the region of the *PHD3* gene queried for DNA methylation (primer sequences and locations described in detail below). After nuclei extraction and a 5 minute DNase I digestion the DNA was extracted and real-time PCR was conducted on an ABI 7000 Sequence Detection System. The accessibility index for each amplicon was then determined by the following formula (accessibility index  =  2^((*C*t Dnase treated) − (*C*t Uncut))^). GAPDH chromatin accessibility was also determined as a positive control for a constitutively expressed gene to control for equivalent DNase digestion between the cell lines examined.

### HRE-Luciferase assay

Cell lines ∼85% confluent in 60 mm dishes were transfected with an HRE-luciferase reporter vector [19] (2.5 µg) and Renilla luciferase (1.5 µg) according to Lipofectamine 2000 transfection reagent protocol. Transfection media was removed after 6 hours and replaced with fresh medium. Cells we then placed under 94% N_2_, 5% CO_2_, 1% O_2_ gas mixture in a hypoxia chamber, or normoxia for 24 hours and then lysed according to the Dual luciferase reporter assay system (Promega, Madison, WI) protocol. Luminescence was measured 3 times per sample using a Tecan SpectraFluor Plus luminometer.

### Adenoviral Transduction

The adenoviral PHD3 construct was a generous gift from Dr. Robert Freeman from Rochester University (unpublished). Briefly, the human PHD3 coding sequence was engineered into the pDC315 vector and contains an N-terminal FLAG tag. PC3 cells were grown to ∼85% confluency and then transduced with 20, 40 or 60 MOI of Ad-PHD3. Approximately 36 hours following transduction, cells were lysed with RIPA buffer and western blotted according to the procedures outlined above.

### Clinical Samples

Clinical prostate tumor samples were received as frozen blocks in OCT. Sections were cut and ground with mortal and pestle. DNA and RNA were extracted in with Qiagen DNeasy Tissue kit and Trizol respectively.

### Methylated DNA Enrichment

Genomic DNA was harvested from cells and tissue using a Qiagen DNeasy Tissue kit. 2 µg of DNA in 120 µl of 10 mM Tris pH 8.0 was sonicated into fragments of approximately 150 bp using a Covaris S2. Fragmentation was done according to the Covaris protocol. 1 µg (60 µl) of sheared input gDNA was used according to the protocol supplied by the MethylMiner kit (Invitrogen). Binding reactions between beads containing methyl-CpG binding domains and sheared genomic DNA were performed at 4°C overnight. Bound DNA was eluted using progressively increasing NaCl concentrations. Eluates were precipitated using NaOAc and EtOH precipitation and resuspended in 60 µl 10 mM Tris pH 8.0. Real time PCR was performed using 1 µl resuspended DNA, SYBR Green master mix and 100 nM Fwd Primer: 5′-GAGCTCCACGACCCGTTTC-3′ and Rev Primer: -5′-GCAGTGGTGGCTTCCCAT-3′ in a 10 µl reaction volume. The kit was validated using samples from human tumor cell lines with know *PHD3* CpG island methylation status as determined by bisulfite sequencing (**Figure S1**).

### Statistical Analysis

Significant differences between groups of data were determined using a t-test for all bar graphs or ANOVA for box plot powered by SigmaPlot 11.0 software. n = 3 was used for each data set unless otherwise noted.

## Results

### PHD3 mRNA is aberrantly silenced in human melanoma, prostate and breast carcinoma cell lines

**Figure 1:**
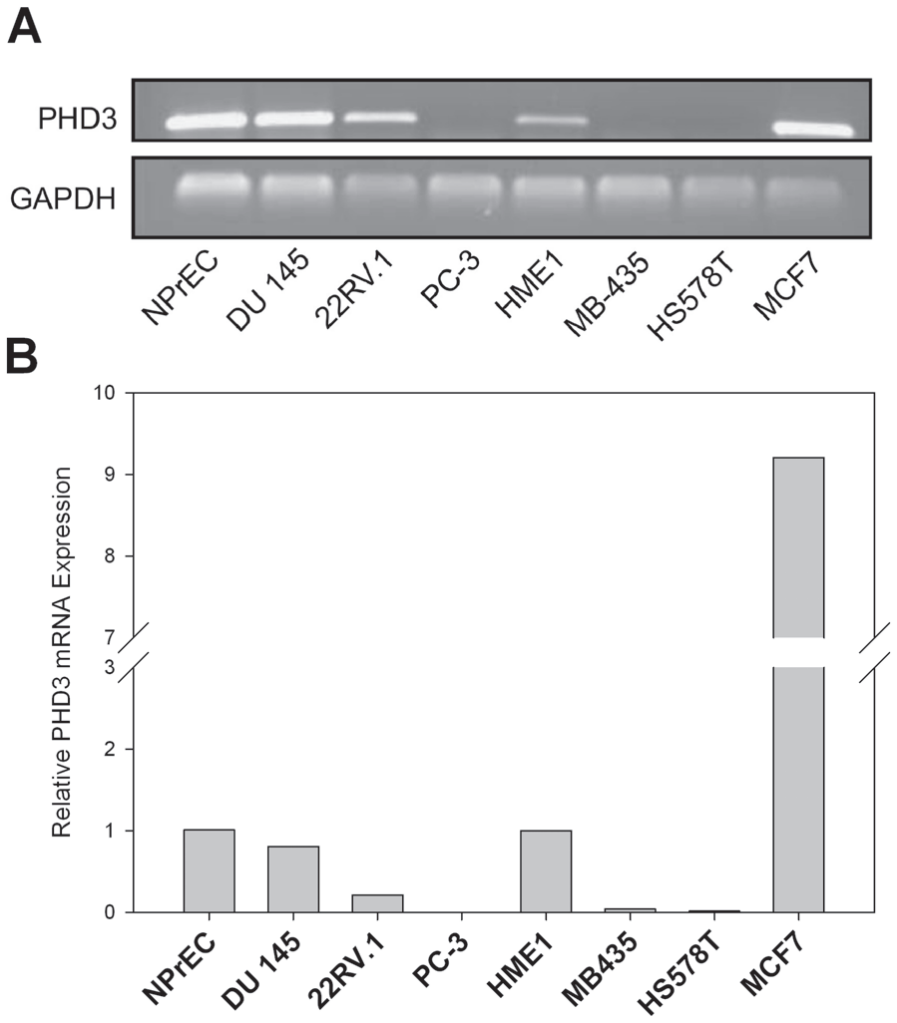
Differential expression of PHD3 mRNA in normal and tumor cell lines of prostate, breast and melanoma origin. Total RNA was extracted from the indicated cell lines. **A**) Expression of mRNA was determined by semiquantitative RT-PCR using specific primers designed to amplify PHD3 and GAPDH. The RT-PCR products were electrophoresed using 1% agarose gel, with ethidium bromide to show bands of expected sizes. GAPDH was used as the loading control. **B**) Quantitative real-time reverse transcription-PCR analysis of *PHD3* was performed with normalization to 18S rRNA gene expression and relative quantities were determined by the DDCt method. The data shown are the *PHD3* gene expression of individual cancer cell lines relative to those of their corresponding normal counterparts (PrEC for prostate and HMEC-hTERT for mammary).

A panel of human carcinoma cell lines was screened for PHD3 mRNA expression. This panel consisted of three prostate cancer cell lines (DU 145, 22RV.1 and PC-3), two breast cancer cell lines (MCF7, HS578T), one melanoma (MDA-MB-435), a non-transformed prostate epithelial cell line (NPrEC) and a non-transformed, immortalized breast cell line (hTERT-HME1). We found that PHD3 mRNA was expressed at different levels varying from abundant to almost undetectable levels as determined by conventional RT-PCR (**Figure 1a**). The prostate cancer cell lines showed decreased PHD3 mRNA expression compared to the normal prostate epithelial cells. A comparison of three prostate cancer cell lines showed that PHD3 is expressed in DU 145 and 22 RV.1, whereas in PC-3, PHD3 mRNA is nearly undetectable. The melanoma cell line, MDA-MB-435 was also found to have very low PHD3 mRNA expression. Among the mammary cell lines, HS578T had much lower PHD3 mRNA than the HME1 mammary epithelial cells and MCF7 cells expressed far more PHD3 mRNA than its normal HME1 mammary epithelial cell counterpart. To confirm and extend the results shown in **Figure 1a**, quantitative real time RT-PCR analysis of PHD3 mRNA expression were conducted and the results are shown in **Figure 1b**. Similarities in expression were found with both the methods used, and PHD3 mRNA expression was nearly undetectable in PC-3, MDA-MB-435 and HS578T cell lines.

### Re-expression of PHD3 with DNA methyltransferase inhibitor 5-Aza-dC

**Figure 2:**
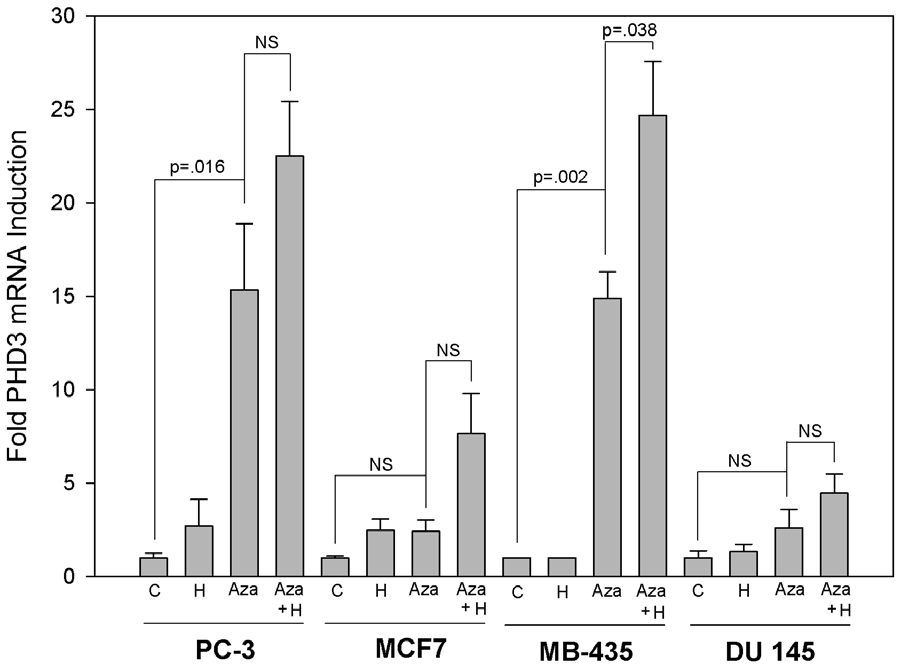
Treatment with 5-Aza-dC triggers re-expression of PHD3. Cell lines representative of PHD3 expressers and PHD3 non-expressers were either not treated (C), treated with hypoxia (H), treated with 5-Aza-dC (Aza), or treated with 5-Aza-dC and then subjected to 1% oxygen (Aza + H) according to the protocol described in Materials and Methods. Real time quantitative PCR was performed to determine the mRNA expression of PHD3. The data were normalized to GAPDH and expressed relative to PHD3 mRNA in the corresponding untreated controls. Error bars  =  SEM. n = 3.

The near absence of PHD3 mRNA expression in a subset of cell lines suggested an epigenetic mechanism might be responsible for their silencing. Unlike genetic mutations that accumulate in cancer, epigenetic modifications are reversible [20]. We hypothesized that if DNA methylation of the PHD3 gene was responsible for its reduced expression, a DNA methyltransferase inhibitor such as 5-Aza-2′-deoxycytidine (5-Aza-dC) should induce its expression. This is in line with 5-Aza-dC's purported ability to re-activate genes previously silenced by DNA methylation in cancer cells [21]. We chose 2 cell lines displaying the most marked decrease in PHD3 expression, MB-435 and PC-3, and 2 cell lines displaying moderate to high basal PHD3 expression, MCF7 and DU 145 for treatment with 5-Aza-dC. When MB-435 and PC-3 cells were treated with 5-Aza-dC, there were significant increases in the PHD3 mRNA expression compared to their respective untreated controls (**Figure 2**). Furthermore, MB-435 cells became responsive to PHD3 mRNA upregulation by hypoxia to a significant degree following hypoxic exposure. Cells that already expressed PHD3 at moderate levels did not respond to 5-Aza-dC by significantly upregulating PHD3. Although this finding suggests that CpG methylation is involved in silencing, further direct queries of epigenetic alterations at this locus were necessary to more deeply address this question.

### The *PHD3* CpG island is aberrantly CpG methylated and displays decreased chromatin accessibility in human carcinoma cells

**Figure 3:**
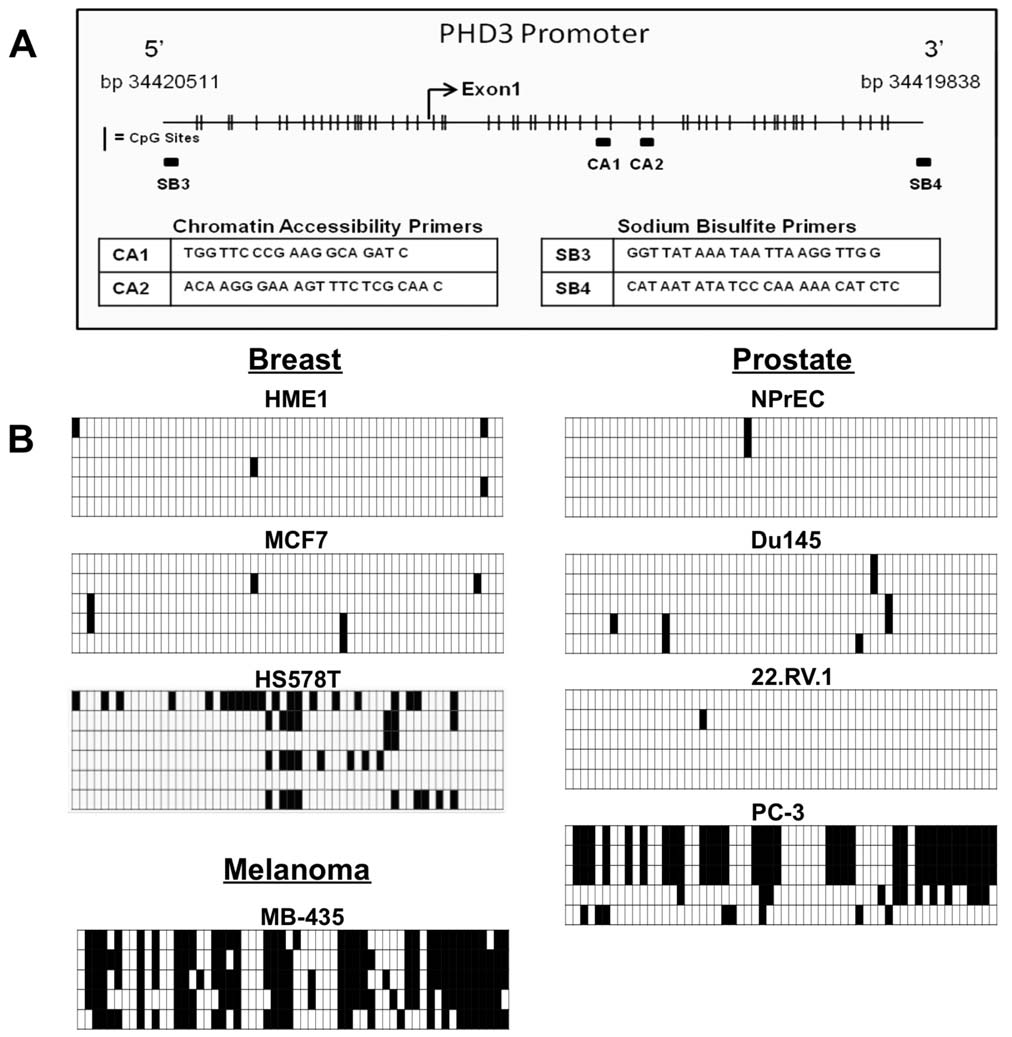
DNA methylation analysis of the *PHD3* 5′-CpG island in human melanoma, prostate and breast carcinoma cell lines. **A**) Schematic representation of the CpG distribution in the 5′-regulatory region of the *PHD3* gene from UCSC Genome Browser coordinates bp34420511-bp34419838. The CpG sites are represented by vertical tick marks, and the beginning of exon 1 is depicted as a bent arrow. The locations of PCR primers used to assess genomic methylation (SB3 and SB4) and chromatin accessibility (CA1 and CA2), respectively. **B**) Bisulfite sequencing was performed on DNA isolated from two non-tumorigenic epithelial cell lines (NPrEC and hTERT-HME1) and six tumor cell lines (DU 145, 22RV.1, PC-3, MB-435, HS578T, and MCF7). Each grid contains at least 5 rows representing the 5 individually cloned and sequenced bisulfite PCR products from the indicated cell lines. Each row contains 58 boxes representing the 58 CpGs in an individual cloned molecule. Open and filled boxes are unmethylated and methylated CpG sites, respectively.

To determine whether *PHD3* gene methylation is present at the CpG island in cell lines that have reduced PHD3 mRNA expression and respond to 5-Aza-dC by upregulating PHD3 mRNA, we utilized sodium bisulfite sequencing to identify methylated CpG sites. **Figure 3A**, illustrates the CpG island in the 5′-end of the *PHD3* gene. The 58 CpG sites in the region analyzed are represented by vertical lines. A putative hypoxia response element (HRE) in the promoter region of the gene is also indicated. We found that these CpGs were highly methylated in the PHD3 negative cell lines PC-3, MB-435, and HS578T (**Figure 3B**). These represent examples of human melanoma, prostate, and mammary carcinoma cells respectively. We also noted that many cell lines appeared to be heterogeneous with respect to *PHD3* promoter methylation status. Within certain cell lines, some clones display high levels of methylation, whereas other have very few to no methylated CpGs. This small population of unmethylated or hemimethylated cells within a cell line may explain our ability to detect very low levels of PHD3 mRNA in cell lines displaying largely methylated *PHD3* CpG islands. Furthermore, areas of CpG methylation in some methylation positive cell lines overlap with a putative HRE in the PHD3 promoter region, which could hinder the ability of PHD3 to be induced upon hypoxic stimuli. In contrast to the positive methylation status in PHD3 negative cells, the CpGs in the *PHD3* CpG island were largely unmethylated in the PHD3 positive cells, NPrEC, DU 145, 22RV.1, HME1, and MCF7.

DNA methylation is typically associated with other alterations to chromatin structure that participate in cell-type specific gene expression patterns. Aberrant cytosine methylation in the 5′-regulatory regions of genes is typically associated with deacetylated histones, and thus a state of DNA that is generally inaccessible to transcription factors and other enzymes that act on DNA, such as polymerase II. This is a mechanism of gene silencing often exploited by cancer cells [22]. In a chromatin accessibility assay, we found the promoter region of *PHD3* in PC-3 cells was resistant to cutting by DNase I when compared to the MCF7 *PHD3* promoter, whereas there was little change in *GAPDH* promoter accessibility between the two cell lines **(Figure S2**). This evidence further supports the hypothesis that *PHD3* promoter methylation and heterochromatin formation are part of the mechanism for reduced expression of PHD3 in these human breast and prostate cancer cell lines.

### PHD3 expression is not induced upon exposure to hypoxia in cell lines containing *PHD3* promoter methylation

**Figure 4:**
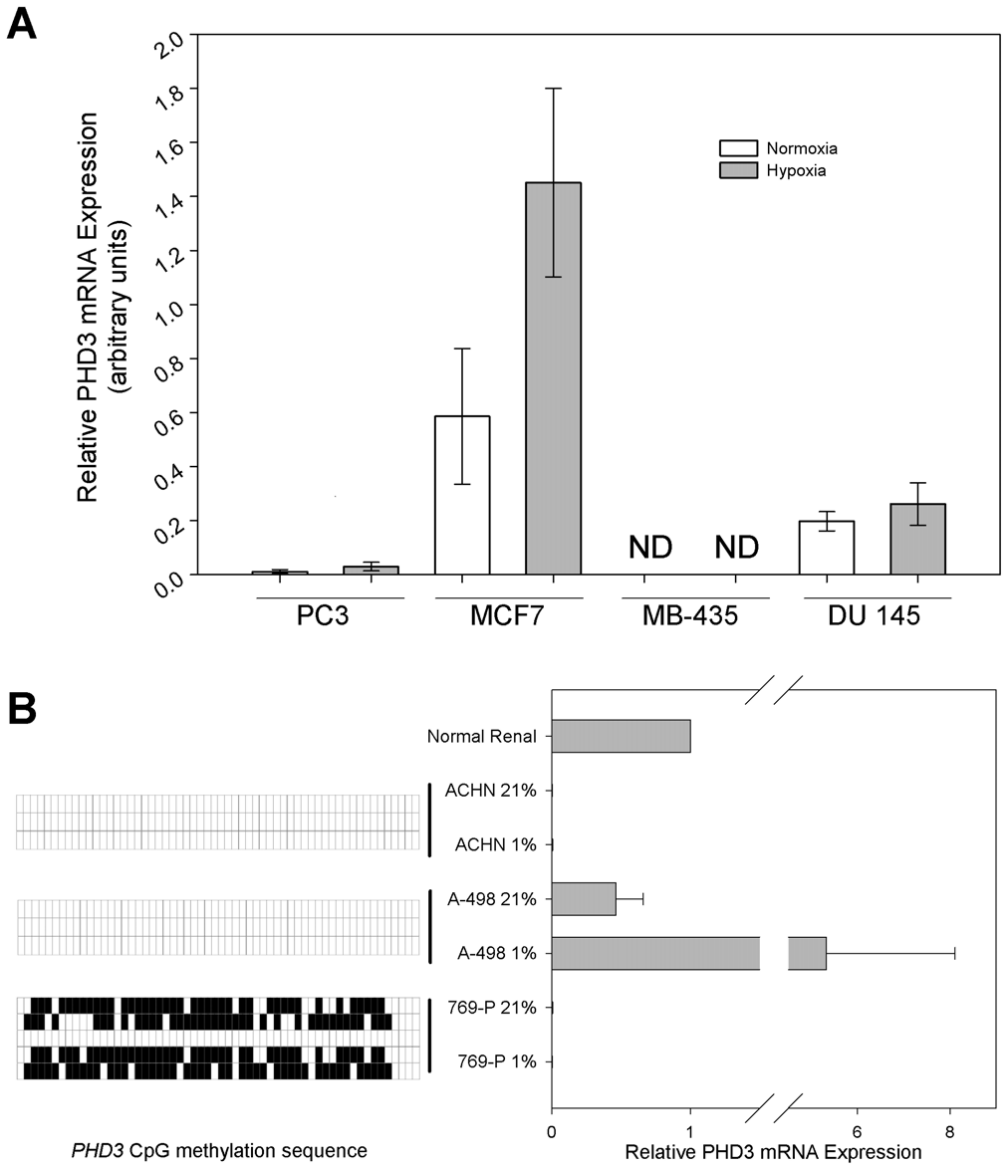
The methylated *PHD3* gene in melanoma, prostate, breast and renal carcinoma cell lines is refractory to induction by hypoxia. **A**) Melanoma, prostate and breast carcinoma cell lines were treated with hypoxia (1% O_2_) or normoxia (21% O_2_) for 24 hours. Total RNA was extracted and converted to cDNA by reverse transcription. Quantitative real-time reverse transcription-PCR analysis of PHD3 was performed with normalization to GAPDH gene expression. Relative quantitation was determined by the DDCt method. ND  =  not detectable. Error bars  =  SEM. n = 3. **B**) Renal clear cell carcinoma cell lines were either untreated or treated with hypoxia as in A). The right panel depicts relative PHD3 mRNA levels compared to mRNA extracted from normal renal tissue. The left panel depicts the methylation status at each of 58 CpG dinucleotides present in the *PHD3* CpG island of the representative renal carcinoma cell lines. Error bars  =  SEM. n = 3.

Unlike *PHD1*, both *PHD2* and *PHD3* genes contain hypoxia response elements, and can be induced by hypoxia by the HIF-1 and HIF-2 transcription factor complex. In the case of PHD3, mRNA and protein expression can be relatively low during normoxic conditions, with marked increases upon hypoxic insult [10]. Therefore, we tested PC-3, DU 145, MB-435 and MCF7 cell lines for their ability to upregulate PHD3 following 24 hours of hypoxia (1% O_2_) (**Figure 4A**). We found cell lines that contained *PHD3* promoter methylation (PC-3, MB-435) failed to appreciably upregulate PHD3 mRNA under these conditions. However, we did note a very small upregulation of PHD3 mRNA in PC-3 cells. This can likely be attributed to the heterogeneity of *PHD3* promoter methylation between specific clones in this cell line (see **figure 3B**). In contrast, PHD3 mRNA was much more prone to upregulation in the unmethylated cell line MCF7. Upregulation of PHD3 in DU 145 cells varied by experiment, and averaged as a non-significant trend toward hypoxic upregulation.

Our discovery of *PHD3* promoter methylation in melanoma, breast and prostate cancer cell lines prompted us to ask whether cell lines from other malignant tissues contained methylation at the *PHD3* locus. Therefore, we further performed real-time PCR and bisulfite sequencing on a panel of 3 human renal cell carcinoma cell lines (A-498, ACHN and 769-P), and on cDNA prepared from normal kidney tissue (**Figure 4B**). We found that ACHN and 769-P cells express nearly undetectable levels of PHD3 mRNA, whereas A-498 expresses levels comparable to normal tissue. Of these cell lines, the PHD3 positive A-498 displayed an unmethylated PHD3 promoter whereas 769-P cells displayed an aberrantly methylated PHD3 promoter. Interestingly though, we did not detect any CpG methylation at the promoter of PHD3 negative ACHN cells, suggesting an alternative mechanism for silencing in this cell line.

### *PHD3* promoter methylation status does not correlate with hypoxia induced HIF-1a protein accumulation or HIF transcriptional activity

**Figure 5:**
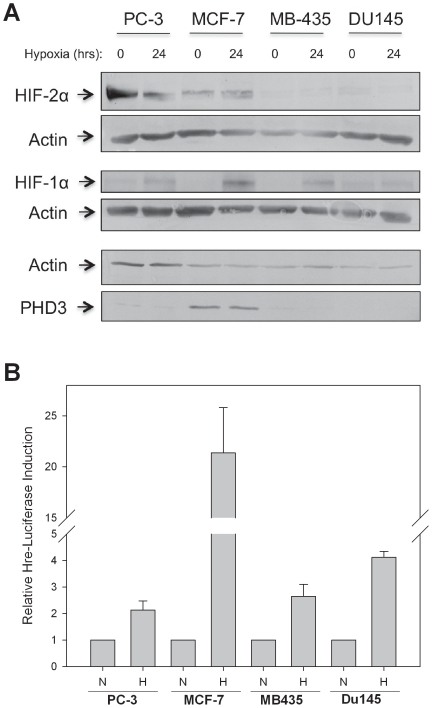
The HIF-1 response is not impaired in prostate and breast carcinoma cell lines that lack *PHD3*. **A**) Melanoma, breast and prostate cancer cell lines with methylated *PHD3* promoters (MB-435, PC-3) and non-methylated promoters (MCF7, DU 145) were subjected to hypoxia (1% O_2_) or normoxia for 24 hours. Thirty micrograms of whole cell lysate was western blotted for the presence of HIF-1α, HIF-2α and PHD3; actin was used as a loading control. **B**) Breast and prostate cancer cell lines were transfected with an HRE-luciferase reporter vector and Renilla luciferase vector and then subjected to hypoxia (1% O_2_) or normoxia for 24 hours. Luciferase activities after 24 hours of hypoxia were determined and are depicted relative to luciferase activities in cells under normoxia. Error bars  =  SEM. n = 3 for MCF7 and PC-3. n = 2 for MB-435 and DU 145.

The presence of *PHD3* promoter methylation in such a broad range of epithelial malignancies suggests that it may be a selective advantage for tumor survival. One hypothesis is that *PHD3* silencing by promoter methylation may allow for an increased HIF transcriptional response during hypoxic conditions. In order to determine whether *PHD3* promoter methylation specifically affects the hypoxia response pathway, we performed western blots on cell lysates from MCF7, PC-3, MB-435 and DU 145 cell lines to compare the HIF protein levels and the HIF transcriptional response to hypoxia (**Figure 5a** **)**. Following 24 hours of hypoxia, HIF-1α protein was upregulated in all the cell lines regardless of PHD3 expression status. We also observed that DU 145 cells appear not to express detectable levels of PHD3 protein. We are unsure whether this is due to limits of detection by our PHD3 antibody, or if DU 145 cells downregulate PHD3 expression by a posttranslational mechanism. Our antibody appears to be specific to PHD3 as transduction of an adenoviral-PHD3 expression vector into PC3 cells produces a band at an identical molecular weight as the band seen in MCF7 cells (**Figure S3**). Interestingly though, MCF7 cells, which do express basal levels of PHD3 mRNA and protein, displayed the largest induction of HIF-1α protein. Thus, HIF-1α protein levels, in general, did not show any correlation with presence or absence of PHD3. We also found HIF-2α to be expressed under normoxic conditions in MCF7. Moreover, MB-435 cells, which express the lowest levels of PHD3 mRNA out of all the cell lines tested appear not to express HIF-2α at an appreciable level. Thus, loss of PHD3 does not appear to be significantly correlated with an accumulation of HIF-1a or HIF-2α levels in these cell lines.

To further investigate the effect of *PHD3* promoter methylation on the transcriptional response of the hypoxia response pathway, we transfected MCF7, PC-3, MB-435 and DU 145 cancer cell lines with an HRE-luciferase reporter construct [19]. Following 24 hours of hypoxia, luciferase activity was measured and plotted relative to luciferase activity in normoxic cells (**Figure 5B**). There was no correlation between *PHD3* promoter methylation status and hypoxic induction of luciferase. The results of this experiment resembled the pattern of HIF-1α protein accumulation seen in **figure 5A**. MCF7 cells showed the largest induction luciferase activity, whereas the other cell lines, which do not express detectable levels of PHD3 protein, were comparable to one another. Although not absolute, these data are highly suggestive that HIF protein stabilization and transcriptional activity is largely independent of PHD3 expression.

### *PHD3* promoter methylation is absent in primary human prostate adenocarcinomas

**Figure 6:**
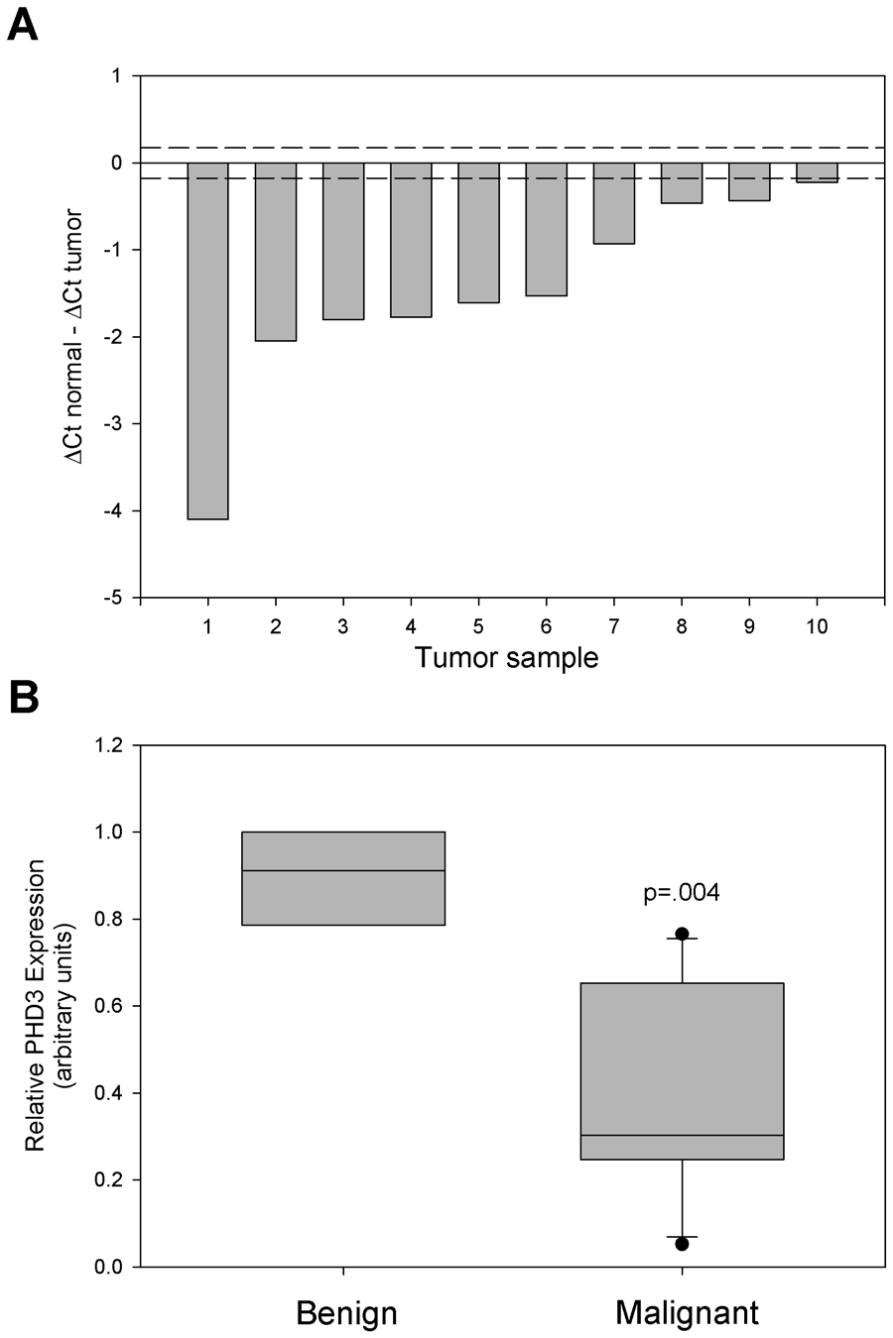
PHD3 mRNA expression is downregulated in multiple primary human prostate cancer specimens. **A**) Total mRNA was isolated from frozen sections of primary human prostate cancer specimens with Gleason scores ranging from 7–9. Quantitative real-time PCR was performed using PHD3 specific TaqMan primer-probe. Relative PHD3 mRNA expression for each tumor sample is represented as (average dCt of n = 3 benign prostate tissue samples) – (dCt tumor sample). Samples were normalized to GAPDH. Dotted lines represent +/− 1 SD for benign tissue PHD3 mRNA expression. **B**) Box plot depicting PHD3 mRNA expression from samples shown in A. p value is based on ANOVA between 3 benign samples and 10 malignant samples.

Recent data published by Hatzimichael et al. suggested that acquisition of *PHD3* promoter methylation may be a relatively common event in certain plasma cell neoplasias [17]. Therefore, we asked whether primary human prostate neoplasias contained methylation at the *PHD3* CpG island. We extracted DNA and RNA from frozen sections of 10 prostate cancer specimens containing a minimum of 70% malignant tissue with a Gleason score ranging from 7–9 as well as 3 benign prostate specimens. Real-time PCR of extracted RNA showed that all 10 tumors contained decreased PHD3 mRNA expression compared to 3 benign tissue specimens (**Figure 6A and B**). Probing for methylated CpGs in select tumor specimens using the MethylMiner kit indicated the possible presence of methylated CpGs at the *PHD3* CpG island in tumor sample 1 (**Figure S4**), which also contained the lowest PHD3 mRNA levels. However, further bisulfite sequencing of 8 clones from tumor sample 1 and tumor sample 4 did not detect any methylated CpGs (data not shown). This could be due to better sensitivity to population average methylation states as compared to the single molecule at a time approach of bisulfite sequencing.

## Discussion

Perturbations in the cellular responses to hypoxia are well known to play a role in the malignant process. Familial mutations in *VHL*, a negative regulator of the HIF-α proteins, results in vascular tumors of the brain, spinal cord and retina, as well as appearance of renal clear-cell carcinomas [23]. PHD proteins play a role upstream of VHL regulation; they hydroxylate HIF-α proteins, creating a binding site for VHL [24], [25], [26]. Thus, it is feasible that deregulation of PHD activity or expression could also contribute to the malignant process. In fact, an absence of PHD3 upregulation following hypoxia has been observed in multiple human cell lines from tumors of the breast, prostate and brain [10], [16]. A recent clinical study of breast tumors containing *BRCA* mutations supports the hypothesis that PHD3 plays an important role in malignancy. This study found a positive correlation between decreased PHD3 expression and a basal phenotype, which is considered a higher grade and more aggressive tumor [27].

Here, we report aberrantly silenced basal mRNA expression of PHD3 in breast, prostate, melanoma and renal cell carcinoma cell lines, and the absence of PHD3 mRNA induction upon hypoxic stimulus. PHD3 expression could be recapitulated in some PHD3 negative cell lines after treatment with 5-Aza-dC, a DNA methyltransferase inhibitor, implicating DNA methylation as a mechanism for the decreased expression of PHD3 mRNA in these cell lines. *PHD3* promoter methylation was verified by sequence analysis of PCR products cloned from bisulfite-treated genomic DNA. We found that among the human cancer cell lines investigated, PC-3, MB-435, HS578T, and 769-P cell lines have hypermethylated *PHD3* CpG islands. Furthermore, the *PHD3* promoter region was more resistant to DNase I in PC-3 cells (hypermethylated *PHD3* promoter) compared to MCF7 cells (hypomethylated *PHD3* promoter). The methylation of the *PHD3* promoter in these carcinoma cell lines appears to be aberrant since insignificant DNA methylation was found in the non-transformed cell counterparts of prostate and mammary epithelial cell lines NPrEC and HME1 respectively. The apparently aberrant *PHD3* promoter methylation status in these cell lines is the likely mechanism, at least in part, for PHD3 transcriptional suppression because DNA methylation is typically associated with a condensed heterochromatin state, and is known to inhibit transcription factor binding to promoter regions of genes [28].

Although all members of the PHD family have the ability to hydroxylate both HIF-1 and HIF-2α, the specificities appear to differ slightly. PHD2 has been reported to play a more pronounced role in the regulation of HIF-1α, whereas PHD3 more strongly affects HIF-2α stability [10]. Therefore, it would seem likely that loss of PHD3 expression by promoter methylation would convey a cellular advantage mediated through increased HIF-1α and/or HIF-2α stability during hypoxia. This could lead to increased expression of VEGF and erythropoietin with subsequent vascular recruitment. Tumors of the breast, skin, kidney and prostate, being solid tumors, would certainly benefit from an increase in vascular supply to hypoxic areas. In fact, there is evidence that cell lines from other solid tumors downregulate PHD3 as well. Henze et al. have shown that several glioma cell lines display little to no PHD3 protein expression during normoxia, with no induction upon hypoxia when compared to other glioma cell lines studied [16]. Our results would predict that a subset of those cell lines have aberrant methylation of the *PHD3* CpG island. Interestingly, HIF-2α protein levels after 18 hours of hypoxia appeared lower in PHD3 non-expressing cells than those in cells expressing relatively high levels of PHD3 [16]. This is the opposite of what we had expected, and may demonstrate the ability of other PHD family members to substitute for the loss of PHD3 expression in regulating HIF-1α and HIF-2α stability. This hypothesis is supported by data from Appelhoff et al., who measured relative protein amounts of PHD isoforms in multiple cell lines. In cell MB-435 and ZR751 cell lines where PHD3 is low or absent, PHD2 protein is greatly elevated.[29]. Similarly, we found that MCF7 cells, which basally express PHD3 at the mRNA and protein level during normoxic conditions, also express relatively high levels of HIF-2α. Taken together, we interpret these findings to suggest that *PHD3* silencing by CpG methylation may not have a significant impact on HIF-1 and HIF-2α protein levels in the cell lines that we tested.

In fact, the results of our study support a mechanism whereby PHD3 silencing by *PHD3* CpG island methylation affects pathways outside of the conventional hypoxic response pathway. When HIF transcriptional activity was measured through a hypoxia responsive HRE-luciferase reporter, we observed nearly equal transcriptional responses to hypoxia in 3 out of 4 cell lines, which included both PHD3 silenced as well as PHD3 expressing cell lines. In MCF7 cells, which express PHD3 at the protein level and do not contain *PHD3* promoter methylation, we observed nearly a 15-fold increase in luciferase induction upon hypoxic treatment. These results suggest that PHD3 expression status does not significantly affect HIF protein stabilization or HIF transcriptional activity through an HRE containing promoter upon exposure to 1% oxygen. Therefore, modulation of an alternative cellular pathway(s) remains an open candidate for mediating the effects of PHD3 loss in malignancies.

Besides the HIF family proteins, other interacting partners of PHD3 have already been discovered. PHD3 is a known player in both neuronal apoptosis and in myoblast differentiation [30], [31] PHD3 also appears to interact with Bcl-2 to induce apoptosis in H9c2 cells in response to doxyrubicin [32].Furthermore, PHD3 has also been reported to destabilize ATF-4 through a novel oxygen-dependant domain on ATF-4 [33]. ATF-4 is involved in the regulation of angiogenesis and metabolism [34]. Thus, upregulation of ATF-4 by a loss of PHD3 could promote cell survival. In addition, PHD3 has also been recently reported to inhibit IKKβ. An increase in IKKβ activity in the absence of PHD3 could confer a growth advantage to cells through an increase NFκB signaling [35]. Constitutive NFκB activity is an important and common event in T- and B-cell derived malignancies [36], [37] and may explain the *PHD3* promoter methylation recently reported in plasma and B-cell neoplasia by Hatzimichael et al. [17]. These interesting prospects related to identification of the downstream targets of PHD3 signaling will undoubtedly become a focus of future investigations.

Our inability to detect *PHD3* promoter DNA methylation in primary human prostate tumors was surprising, however our results are supported by a recent study by Huang et al. [38], who screened 168 invasive breast carcinomas and did not find evidence of *PHD3* DNA methylation using melting curve analysis of bisulfite converted DNA. We cannot rule out the possibility that a low level of semi-methylated *PHD3* CpG islands are present in their samples as the “intermediately methylated” controls were not truly intermediately methylated, but rather were mixtures of 100% methylated DNA with 0% methylated DNA. Nonetheless, it seems clear that a large proportion of primary epithelial tumors does not contain a high degree of *PHD3* promoter methylation, and may not be the ideal specimens for detection of methylation at this locus. Given PHD3′s purported ability to negatively regulate the NFκB pathway [30], [35], and the widely reported involvement of NFκB in cell migration and metastasis [39], [40], future studies on *PHD3* CpG island methylation in clinical samples of metastatic disease as opposed to primary tumors may yield more positive results.

Here, we are the first to report DNA methylation of the *PHD3* CpG island in solid tumor cell lines derived from diverse cell types. *PHD3* methylation in carcinoma cells was associated with their inability to appropriately upregulate PHD3 mRNA upon exposure to hypoxia. We are also the first to show evidence that this aberrant expression fails to correlate with an increase in HIF protein accumulation and transcriptional activity upon exposure to hypoxia in the cell lines examined. The presence of *PHD3* promoter hypermethylation and PHD3 silencing in such a wide range of cancer types suggests this might be a common event that elicits a selective advantage for tumors. Our data suggest that at least in some cell lines, the nature of this advantage may extend beyond hypoxia resistance. Furthermore, the selective event may occur during or after the process of invasion/metastasis, as we and others have not found evidence of methylation in primary solid tumors [38].

## Supporting Information

Figure S1 Validation of methylated DNA enrichment as a tool for detecting methylated CpG regions in human genomic DNA. A) Genomic DNA from human melanoma and breast cancer lines was enriched for methylated CpG dinucleotides at the PHD3 CpG island using the MethylMiner kit, followed by quantitative real-time PCR analysis using PHD3 CpG island-specific PCR primers. S  =  supernatant, representing unmethylated DNA. E1 - E5 represents elutions with 200, 650, 1100, 1550, 2000 mM NaCl respectively. Input  =  1/60th total input DNA. Total amounts of eluted DNA from each fraction are represented as a fraction of input (left). Methylated CpG sequences from bisulfite-converted DNA in the corresponding cell lines are depicted for comparison (right). B) Control 100% methylated and 0% methylated oligos supplied by the MethylMiner kit were subjected to the MethylMiner protocol. S  =  supernatant, representing unmethylated DNA. E1-E5 represent elutions with 200, 650, 1100, 1550, 2000 mM NaCl respectively. Identical volumes of DNA from each eluate were subjected to quantitative real-time PCR using primers supplied by the MethylMiner kit. DNA content in each fraction is represented as arbitrary units.
(10.36 MB TIF)

Figure S2 The methylated PHD3 gene in non-expressing cells is maintained in a less accessible state than the non-methylated PHD3 gene in expressing cells. A) Nuclei from PHD3-positive MCF7 and PHD3-negative PC-3 carcinoma cell lines were isolated and enzymatically restricted with DNase I. Primers CA1 and CA2 (see Fig. 3A) were used for quantitative real-time PCR (right panels) to amplify a region also assessed for cytosine methylation. Accessibility indices (left panels) were calculated as follows: AI  =  2((Ct DNase treated) - (Ct Untreated)). B) GAPDH accessibility indices were simultaneously assessed as a control for a constitutively expressed gene in both cell lines.
(3.00 MB TIF)

Figure S3 PHD3 antibody specificity. PC3 cells were transduced with an increasing MOI of adenoviral-PHD3 vector. Western blot using Novus100–139 antibody co-incubated with anti-actin antibody indicated an approximately 27 kDa band in MCF7 cells that migrates at the same molecular weight as a band present in PHD3 transduced PC3 cells.
(3.00 MB TIF)

Figure S4 Methylated DNA enrichment of genomic DNA isolated from primary human prostate cancer. A) Total genomic DNA was isolated from frozen sections of 7 malignant prostate cancers and 3 benign prostate samples and subjected to the MethylMiner protocol. Tumor sample number corresponds to samples shown in figure 6A. S  =  supernatant, representing unmethylated DNA. E1-E4 represents elutions with 300, 550, 800, and 2000 mM NaCl. PHD3 CpG island DNA content in each fraction is represented as a fraction of total PHD3 CpG island DNA present in input.
(8.41 MB TIF)
