# Supplementary figures and images for: Aberrant Promoter CpG Methylation Is a Mechanism for Impaired PHD3 Expression in a Diverse Set of Malignant Cells
Source: PLoS One. 2011 Jan 28;6(1):e14617. doi: 10.1371/journal.pone.0014617 (PMC3030558; doi:10.1371/journal.pone.0014617)

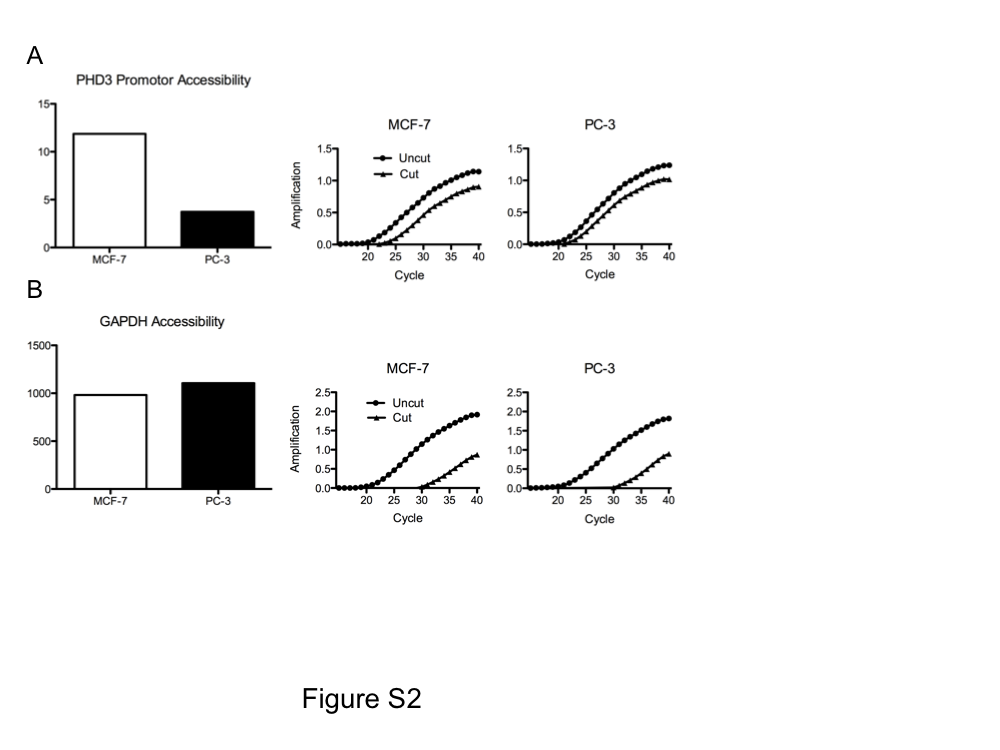

Supplement: Figure S2 — The methylated PHD3 gene in non-expressing cells is maintained in a less accessible state than the non-methylated PHD3 gene in expressing cells. A) Nuclei from PHD3-positive MCF7 and PHD3-negative PC-3 carcinoma cell lines were isolated and enzymatically restricted with DNase I. Primers CA1 and CA2 (see Fig. 3A) were used for quantitative real-time PCR (right panels) to amplify a region also assessed for cytosine methylation. Accessibility indices (left panels) were calculated as follows: AI = 2((Ct DNase treated) - (Ct Untreated)). B) GAPDH accessibility indices were simultaneously assessed as a control for a constitutively expressed gene in both cell lines. (3.00 MB TIF) [file pone.0014617.s002.tif]

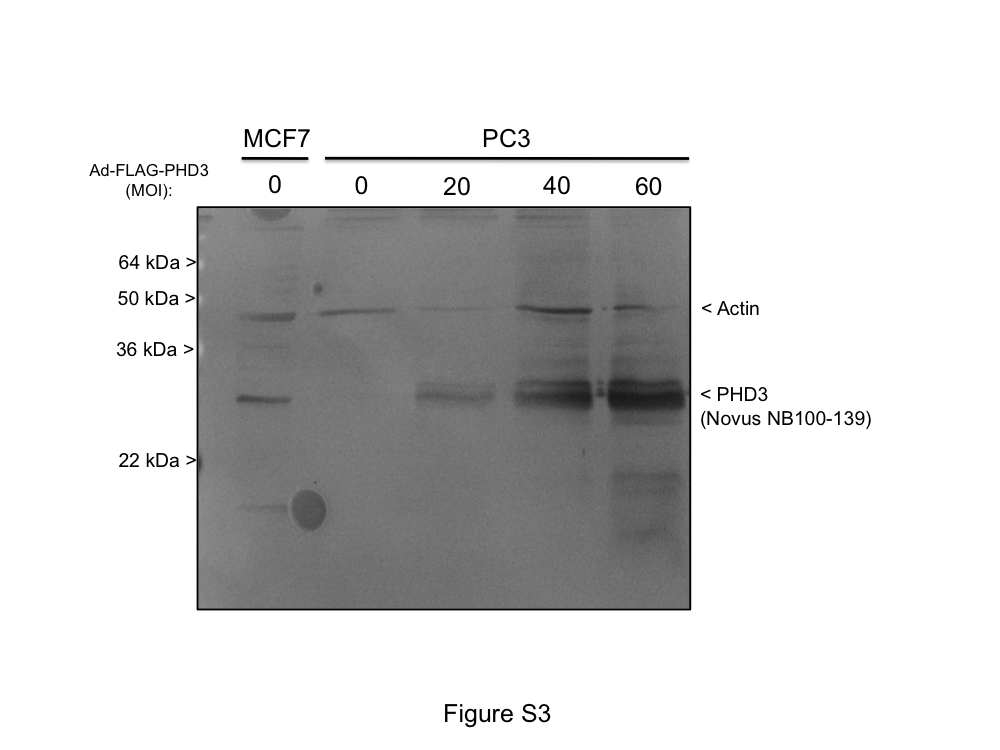

Supplement: Figure S3 — PHD3 antibody specificity. PC3 cells were transduced with an increasing MOI of adenoviral-PHD3 vector. Western blot using Novus100–139 antibody co-incubated with anti-actin antibody indicated an approximately 27 kDa band in MCF7 cells that migrates at the same molecular weight as a band present in PHD3 transduced PC3 cells. (3.00 MB TIF) [file pone.0014617.s003.tif]
